# Variation and correlations between sexual, asexual and natural enemy resistance life-history traits in a natural plant pathogen population

**DOI:** 10.1186/s12862-019-1468-2

**Published:** 2019-07-12

**Authors:** Elina Numminen, Elise Vaumourin, Steven R. Parratt, Lucie Poulin, Anna-Liisa Laine

**Affiliations:** 1 Department of Biosciences, University of Helsinki, Viikinkaari 1, PO Box 65, FI-00014 Helsinki, Finland; 2 Université de Nantes, Faculté des Sciences et des Techniques, Laboratoire de Biologie et de Pathologie Végétales (LBPV), EA 1157, SFR 4207 QUASAV, 2, rue de la Houssinière, BP 92 208, F-44322 Nantes Cedex 3, France; 3 University of Liverpool, Institute of Integrative Biology, Liverpool, L69 3BX UK; 4 Department of Evolutionary Biology and Environmental Studies, University of Zurich, Winterthurerstrasse 190, CH-8057 Zurich, Switzerland

**Keywords:** *Ampelomyces* spp., Diversity, Epidemiology, Fitness, Host-pathogen interaction, *Plantago lanceolata*, *Podosphaera plantaginis*

## Abstract

**Background:**

Understanding the mechanisms by which diversity is maintained in pathogen populations is critical for epidemiological predictions. Life-history trade-offs have been proposed as a hypothesis for explaining long-term maintenance of variation in pathogen populations, yet the empirical evidence supporting trade-offs has remained mixed. This is in part due to the challenges of documenting successive pathogen life-history stages in many pathosystems. Moreover, little is understood of the role of natural enemies of pathogens on their life-history evolution.

**Results:**

We characterize life-history-trait variation and possible trade-offs in fungal pathogen *Podosphaera plantaginis* infecting the host plant *Plantago lanceolata*. We measured the timing of both asexual and sexual stages, as well as resistance to a hyperparasite of seven pathogen strains that vary in their prevalence in nature. We find significant variation among the strains in their life-history traits that constitute the infection cycle, but no evidence for trade-offs among pathogen development stages, apart from fast pathogen growth coninciding with fast hyperparasite growth. Also, the seemingly least fit pathogen strain was the most prevalent in the nature.

**Conclusions:**

We conclude that in the nature environmental variation, and interactions with the antagonists of pathogens themselves may maintain variation in pathogen populations.

**Electronic supplementary material:**

The online version of this article (10.1186/s12862-019-1468-2) contains supplementary material, which is available to authorized users.

## Background

Genetic and phenotypic variation in pathogen populations determines their evolutionary potential and therefore is an important factor driving the emergence, spread, and persistence of disease within their host populations [15, 24]. Until recently, our ability to quantify levels of genetic variation within pathogen populations was restricted by the scarcity of suitable genetic markers [16]. With the development of more specific tools, molecular tracking of pathogen strain variation, and thus disease dynamics, has become possible across a range of spatial and temporal scales [8, 32, 44, 50]. Subsequently, we now know that variation in pathogen populations is ubiquitous across different pathosystems [43]. However, little is known about the mechanisms that maintain this variation in pathogen populations.

A longstanding hypothesis in ecology is that multiple life-history strategies may coexist within populations because differences in their fitness payoffs prevent any one strategy from reaching fixation [37, 38]. Trade-offs in pathogens are of particular interest because they may constrain the evolution and epidemiology of diseases. There is experimental evidence for a trade-off between parasite growth and reproduction, presumably because environments that are optimal for growth may be suboptimal for reproduction and vice versa [5]. Similarly, there is support for a trade-off between parasite virulence and reproduction [14, 47]. However, it should be noted that such trade-offs are not pervasive, and the strength and even shape of life-history correlations can vary according to host genotype, and the abiotic environment [23]. Thus, variation in life-history may promote the maintenance of genetic variation. However, one of the key challenges in studying pathogen life-history variation stems from the inconspicuous nature of the pathogenic life-style. For most pathogens, any attempt to quantify the key life-history stages that lead to infection and transmission require destructive sampling. Moreover, the sexual stage is notoriously difficult to study, and thus for most pathogens we lack sufficient insight into the production and maturation of sexual structures [42]. This is non-trivial given that the sexual stage plays a key role in generating variation in pathogen populations [25], and is also ecologically important because many pathogens survive unfavorable conditions via sexually produced resting spores [2, 35].

To obtain a full understanding of pathogen life-history, it is also important to take into account that a pathogen interacts not only with its host but also its own natural enemies such as predators or hyperparasites (reviewed in [29]); parasitic organism that uses another parasitic organism as a host. Empirical and theoretical studies have found that hyperparasites can fundamentally affect the expression and evolution of key pathogen life-history traits [10, 20, 45, 54], and thus impact pathogen dynamics [3, 18, 36, 49]**.** To date, several studies have demonstrated that pathogen strains vary in their sensitivity to hyperparasites [9, 30]. However, whether investment in resistance to hyperparasites has a cost in other life-history stages has not been explored.

Here, we investigate how pathogen life-history traits vary among seven distinct strains of a powdery mildew fungal pathogen, and whether there are trade-offs between these traits. Our study is able to take into account two important pathogen life-history stages: asexual (i.e life-history traits linked to the growth) and sexual (i.e life-history traits linked to the production of overwinter survival structures), which is relatively uncommon as well as pathogen sensitivity to its common hyperparasite [30]. Moreover, we combine large-scale field epidemiological surveys with multi-locus genetic data to determine whether different life-history strategies relate to the prevalence of strains in the wild. We use the well-characterized pathosystem *Podosphaera plantaginis* – *Plantago lanceolata* (powdery mildew – ribwort plantain) in the Åland Islands (Finland) to answer these questions. Given that this pathogen completes its entire life-cycle on the surface of the host plant, we are able to monitor the life-history traits visually in a non-destructive manner.

We find significant variation among seven pathogen strains in their life-history traits that constitute the infection cycle, with no evidence for trade-offs among pathogen development stages. However, we show that fast growth comes at a cost of being more sensitive to infection by the fungal hyperparasite *Ampelomyces spp*. We do not find a link between specific pathogen traits under laboratory conditions and high prevalence in nature, which suggest that pathogen success in the wild is not a the product of outright growth rate alone.

## Methods

### The study system and pathogen life-history

**Fig. 1:**
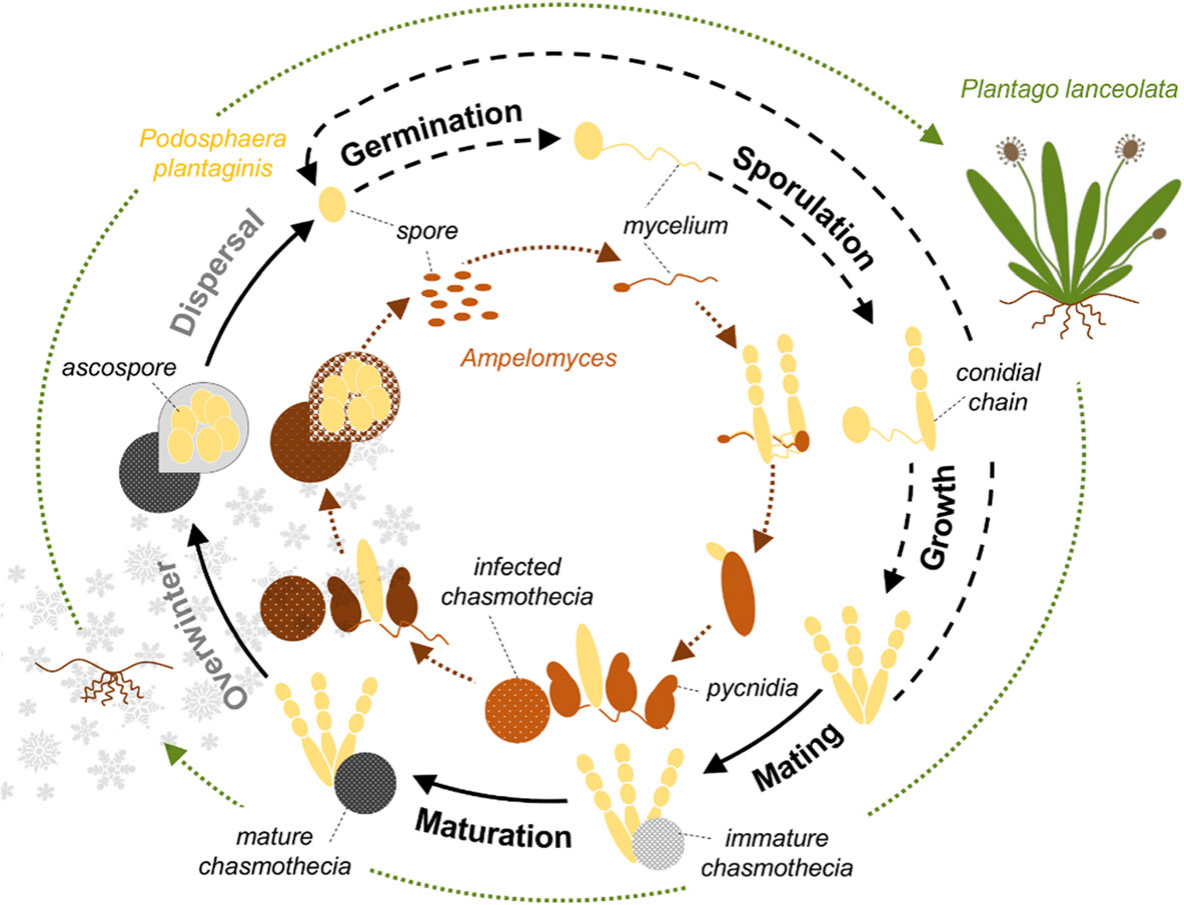
Powdery mildew life-cycle (*Podosphaera plantaginis*) and its hyperparasite *Ampelomyces* spp. on ribwort plantain (*Plantago lanceolata*). The powdery mildew life-cycle is shown in black and the *Ampelomyces* life-cycle in brown. The asexual part of the powdery mildew cycle is indicated by dashed (black) arrows while the sexual part is indicated by solid arrows. The powdery mildew life-cycle begins with the arrival of a spore on a susceptible host leaf. Then the mycelium grows and clonal spores (conidia) are produced. During the growing season, the pathogen is transmitted passively among hosts by wind dispersal of clonal spores. The pathogen survives winter as resting spores (chasmothecia) which contain sexually produced ascospores. Ascospores germination initiates a new epidemic in spring. The *Ampelomyces* life-cycle is strongly dependent on that of powdery mildew as it forms pycnidia within powdery mildew tissues. Hyperparasite spores are released from mature pycnidia and dispersed by rain-splash to nearby powdery mildew colonies

We focused our study on the powdery mildew*, Podosphaera plantaginis* (*Erysiphales*, Ascomycota), which is an obligate fungal pathogen naturally infecting host plant *Plantago lanceolata*, the ribwort plantain. The life-cycle of the powdery mildew begins with the germination of a spore on a susceptible host resulting in lesions where clonal spores (conidia) are produced. During the growing season, these spores are transmitted by wind to new hosts. To survive the winter, resting structures (chasmothecia) are produced that contain sexually generated ascospores, which initiate new infections in the following spring (Fig. 1). *Podosphaera plantaginis* is capable of haploid selfing and hence outcrossing with another strain is not necessary for its overwinter survival [48].

Since 2001, every year in early September all known approximately 4000 *P. lanceolata* populations in the Åland islands, Southwest of Finland, are surveyed for the presence of *P. plantaginis* [27]. At this time of the year the fungus is clearly visible on host leaves as white-greyish mycelial growth with conspicuous black resting spores. Infected leaf samples are collected from each infected site for subsequent genotyping. Fourteen years of epidemiological data have shown that this pathogen persists regionally as a highly dynamic metapopulation [19, 22]. Genotyping has revealed considerable genetic diversity in this pathogen metapopulation, with most of the strains found in only one or few localities with a small subset of strains being common across multiple host populations [50].

Recently, it was reported that pathogen populations in the Åland Islands are frequently infected (77%) by a fungal hyperparasite *Ampelomyces* spp. (Ascomycetes) [30]. *Ampelomyces* is an obligate hyperparasite, and is thus strongly dependent on the life-cycle of its powdery mildew host (Fig. 1). Laboratory experiments have shown that *Ampelomyces* infection decreases the growth of conidia and the production of chasmothecia by *P. plantaginis* [31], and hyperparasite infection in nature is associated with decreased overwinter survival of the powdery mildew [49]. Several studies have shown that *Ampelomyces* infection can also curtail epidemics of other powdery mildew pathogens under greenhouse conditions [1, 33], and thus can negatively impact on pathogen fitness. *P. plantaginis* strains have been shown to vary in both qualitative and quantitative susceptibility to *Ampelomyces* infection [30], but how this variation correlates with other mildew life-history traits has not been studied before. The mode of *Ampelomyces* overwintering in *P. plantaginis* remains still unknown, though in other powdery mildew species it has been shown to survive as pycnidia and as hyphae on the mildew lesion or inside the chasmothecia which are the overwinter structure of the powdery mildew [41].

### Host and pathogen material for the experiment

We first conducted laboratory inoculation experiments to measure possible trade-offs between successive life-history traits that constitute both the transmission phase during epidemics – time to germination, time to sporulation and amount of spores produced – as well as between the production of sexual resting spores of *P. plantaginis*. We also quantified the sensitivity of pathogen strains to the *Ampelomyces* hyperparasite in a similar experiment. Altogether the data that are analyzed here come from three distinct experiment blocks, where the third block is subdivided into inoculations with and without the hyperparasite. All the measurements were obtained at the single-leaf level, each experiment constituting 4–20 replications of such single-leaf inoculations for each pathogen strain. The subsequent statistical modelling is also carried out at the single-leaf level, also when the hyperparasite infection progress is characterized, as the pathogen infection stages can be observed from the same leaf. Since the experiments only differ in their onset time and the same plant genotype was used for the inoculations, it makes sense to analyse the data jointly. For more details on the experimental setup, see Chapter 1 Figure S1 and Tables S1-S5 presented in the Additional file 1.

We focused our experiment on seven *P. plantaginis* strains (*i1*, *876–1*, *4000*, *330*, *40A*, *747–4* and *7.4.3301*) whose performance was measured on a broadly susceptible host genotype (490–15, [40]). The strains were obtained as part of routine sampling of the *P. plantaginis* metapopulation in September 2015, being collected as infected leaves, each from a different host plant, and placed on moist filter paper inside Ø 9 cm Petri dishes. Pure strains were obtained by three consecutive single-colony transfers onto healthy leaves [26]. The fungal strains were maintained on detached *P. lanceolata* leaves in Petri dishes in a growth chamber at + 20 °C with 16 L/8D photoperiod. Successive inoculations were carried out to obtain enough spore material for the experiments. The strains were genotyped with a 19 SNP panel [50] to determine their frequency in the natural metapopulation and to confirm that they consisted of a single pathogen strain. The genotyping protocol is described in detail in Chapter 2 of the Additional file 1 (see Table S6 for details). The studied strains have been shown to vary in their prevalence in the wild along a gradient, from colonizing 4 to more than 60 distinct locations (Figure S2 in Additional file 1 Chapter 3).

### Measuring asexual and sexual pathogen life-history stages

To measure the asexual and sexual pathogen life-history stages, we carried out inoculations in which host leaves were placed on moist filter paper in Ø 9 cm Petri dishes, and conidial spores from an infected leaf were gently brushed with a fine paint brush over the entire surface of the healthy leaf. Colonies of similar age and size (*c*. 1.0 cm Ø) were used for the inoculations in order to obtain as similar spore densities as possible. Spores for each inoculation came from a distinct colony from a distinct plant leaf. This procedure has been shown to produce repeatable infection outcomes for unique pathogen strains [21]. While we did not quantify exact spore dose on each colony used for the inoculations, extensive past work on this pathogen has demonstrated that strain level differences are typically seen as differences in the size and number of lesions rather than in the number of spores supported by each lesion (A.-L. Laine, *pers. comm.*) Each inoculation was replicated at least four times and the inoculations were distributed across three experiment blocks as it would not have been possible to score all the infections at the same time (Additional file 1 Chapter 1). Inoculated leaves inside the Petri dishes were kept in a growth chamber at + 20 °C with a 16 L/8D photoperiod. The resulting infections were observed daily with a dissecting microscope starting at 1 day post inoculation (DPI) until at least 23 DPI. Powdery mildew growth was scored using “Bevan’s scale” (adapted from [6]), ranging from 0 to 4 (0: no mycelium, 1: mycelium only, 2: mycelium and sparse sporulation visible only under a dissecting microscope, 3: abundant sporulation and lesion size < 0.5cm^2^, 4: abundant sporulation and lesion size > 0.5cm^2^, for a schematic version of the scale see Additional file 1 Chapter 1: Figure S1). We used these measures to quantify time to germination (measured as the number of days it takes for a spore landing on a leaf to form the first hyphae), time to sporulation (measured as the number of days it takes for the established mycelium to produce conidial spores) and the amount of spores produced. We measured the reproductive success by documenting the time of first appearance of chasmothecia, their maturation as well as their abundance at 23 DPI on a 0 to 5 scale (0: no chasmothecia, 1: < 10, 2: [10;30], 3: [30;60], 4: [60;200], 5: > 200). For an illustration of the measured traits, see Figures S3 and S4 in Chapter 4 of the Additional file 1, and for basic summary statistics of them, see Table S7 in Chapter 5 of the Additional file 1.

### Pathogen sensitivity to the hyperparasite

To quantify variation in the seven *P. plantaginis* for sensitivity to the hyperparasite we conducted a second inoculation experiment in which we challenged each strain with a single *Ampelomyces* isolate collected from a *P. plantaginis* population Åland in September 2014 (allopatric to any of the seven pathogen strains). Prior to experimentation *Ampelomyces* was maintained as pure culture on custom agar media [30]. Twenty detached *P. lanceolata* leaves were inoculated with each powdery mildew strain as described above, then at 8 DPI, 70 μl ± 2 of *Ampelomyces* spore suspension in filter-sterilised H_2_O was aspirated onto each powdery mildew-infected leaf. Spore suspensions were obtained by scraping the surface of four-six week old *Ampelomyces* culture plates into filter-sterilized H_2_O. Spore suspensions were counted with a haemocytometer and then diluted to (1 × 10^6^ spores/ml). Established *Ampelomyces* infections produce distinctive, brown spore structures within their host’s conidia, called pycnidia (Fig. 1). Leaves were observed daily under a dissecting microscope for the appearance of these pycnidia, starting at one day post *Ampelomyces* inoculation until the 23rd day post powdery mildew inoculation. *Ampelomyces* infection severity was scored with an adapted version of the scale reported in Falk et al. [13]: A0: no pycnidia observed, A1: 1–20 pycnidia in each *Ampelomyces* cluster appearing, and A2: either 21–50+ pycnidia in each powdery mildew lesion or between 30 and 50% of powdery mildew covered (for a schematic version of the scale see Figure S4 in Additional file 1). This scale controls for the different amounts of powdery mildew tissue available for the hyperparasite to infect, i.e. small powdery mildew lesions can still support a level A2 of hyperparasite infection even if there is not enough tissue to produce ~ 50 pycnidia (this has been also statistically tested a posteriori on our samples, H0: No impact of the mildew lesion size on the *Ampelomyces* load, χ^2^ = 2.61, *p-value: 0.11*). For each powdery mildew strain, 20 *Ampelomyces*-negative control leaves aspirated with H_2_O were included in the experiment to ensure there is no cross-contamination of *Ampelomyces* between leaves, and to ensure that the powdery mildew stocks used were viable.

### Statistical analyses

#### Variation in developmental rates of powdery mildew strains and their sensitivity to the hyperparasite

We analyzed variation in the speed to germinate, sporulate, produce chasmothecia, the time it takes from first sporulation to first chasmothecia and the time it takes from first chasmothecia to mature chasmothecia (Fig. 1) using survival models as implemented in R-package survival [46]. We considered Cox Proportional Hazards model [12], in which the hazard of an event of interest is defined as:

$$ h(t)={h}_0(t)\mathit{\exp}\left(\eta \right), $$

where *h*_0_(*t*) is the baseline rate of the event happening as a function of time, and the predictors are assumed to have a multiplicative effect on *h*_0_(*t*) via the linear predictor *η*. All survival models had strain identity and experiment block as fixed effects, and their significance as predictors was concluded using pairwise (anova) model comparisons. When analysing differences in the developmental rates between the strains, we base our considerations in the confidence intervals obtained for each strain.

To quantify variation in *P. plantaginis* sensitivity to *Ampelomyces* hyperparasite, we analysed the time it takes for the *Ampelomyces* to reach states A1 and A2, and the time it takes for the *Ampelomyces* to develop from powdery mildew sporulation to state A2, with strain identity and as a fixed effect. The experiment block was omitted from these analyses, as all hyperparasite data came from the same block. Additionally, to account for the possibility that powdery mildew infection state determines the hyperparasite infection success, we considered a model in which the infection state at the day of hyperparasite inoculation (day 8) was considered as a predictor.

#### Analyses of infection severity

To assess strain-related differences in infection severity (Bevan score at day 15 and the abundance of the chasmothecia produced), we utilized ordinal regression models using the function *clmm2()* in R-package *ordinal* [11] with strain identity and experiment block as fixed effects. This approach allows regressing the exploratory variables of interest to a categorical response variable with natural ordering.

#### Relationships between the life-history traits

We analysed the relationships between pathogen life-history traits (positive or negative) and whether these differ among the strains. For this purpose, we modeled all pairwise correlations between the different traits (event times or abundance measures) with strain identity, life-history trait, as well as the experiment identity. The model structure was that of either ordinal regression or survival model depending on the nature of the response variable; whether it was a measure of abundance or timing of an event, respectively.

#### Relationships to the metapopulation prevalence

When relating the measured life-history traits with metapopulation prevalence, we performed Spearman rank correlation on the observed prevalence of the similar multilocus genotype profile within the wild and the mean measured trait of the corresponding strain.

## Results

### Among strain variation in developmental rates

Pairwise model comparisons (Tables S8 and S9 in Additional file 1 chapter 6.1) confirmed that both strain identity and experiment identity were significant predictors for predicting timings of all pathogen infection stages, apart from one single case. When further assessing variation for the timing of key life-history traits among our seven pathogen strains, we considered the estimated relative rates from the fitted survival models, and their corresponding 95% confidence intervals. In all results below, we infer significant differences between two strains in a given rate whenever the corresponding 95% confidence intervals do not overlap. This is a statistically conservative way to assess the main signals on between- strain differences. Thus the arbitrarily chosen baseline strain (330) is out from these considerations, as it constitutes a reference rate, to which other strains are compared to.

**Fig. 2:**
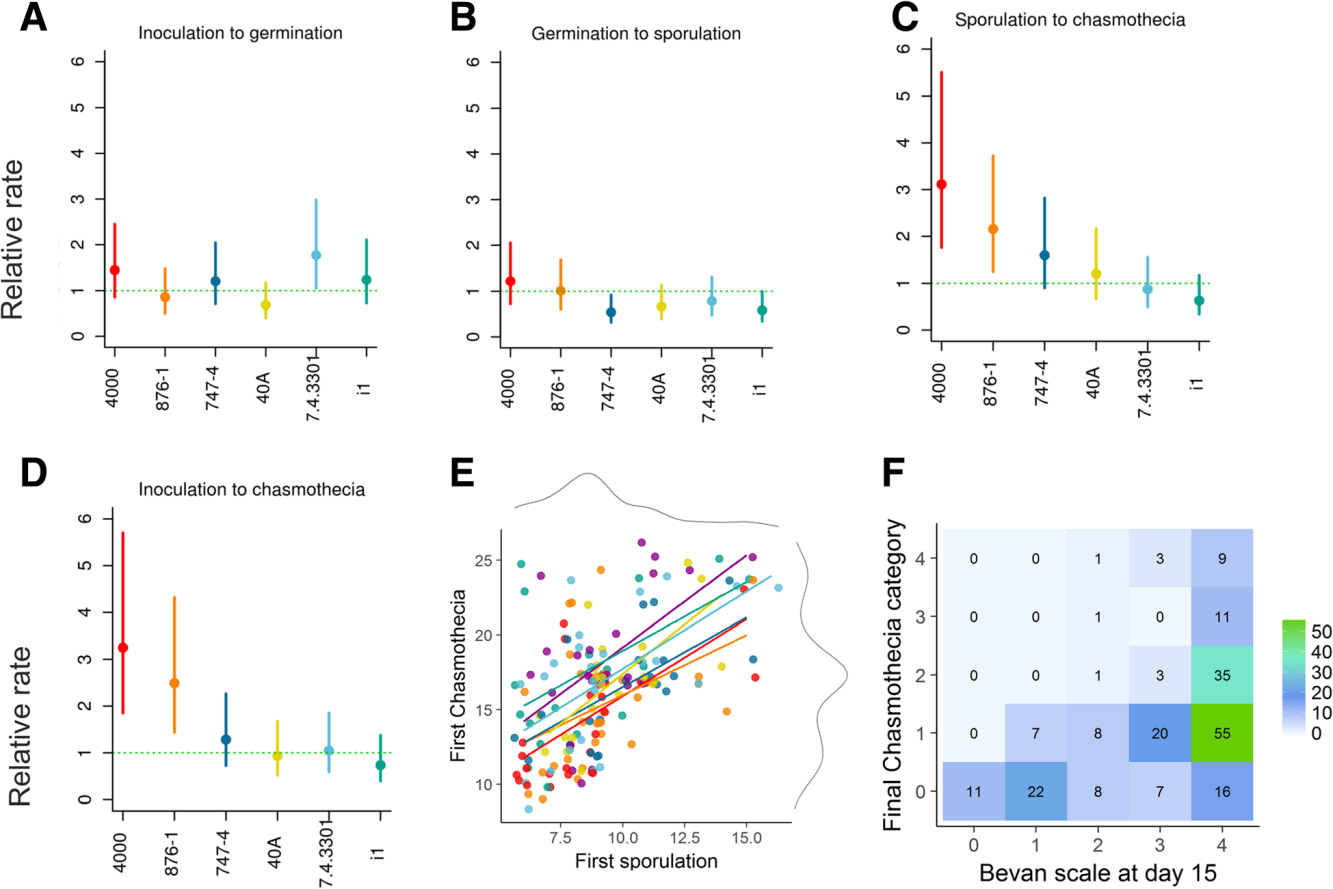
The distributions of the studied powdery mildew life-history traits. Panels **a-d** show the estimated powdery mildew relative rates of reaching different infection stages and their 95% confidence intervals for the individual strains, where strain *330* is considered as the baseline factor (arbitrary). Panel **e** shows that the time to sporulation predicts the time of chasmothecia production (grey lines showing the marginal distributions), both in days. Panel **f** highlights that the Bevan scale at day 15 predicts final chasmothecia abundance

Mean time to germination varied between 2.53 and 3.32 (days) among strains, but there was no evidence for this variation being significant (Fig. 2a). Mean time to sporulation varied from 8.57 and 11 days among strains. The fastest strain to sporulate, strain *4000,* had an estimated relative rate of 1.76 with 95% confidence interval [1.04, 2.96], indicating faster sporulation than the baseline rate (since the confidence interval does not include 1). The slowest strain to sporulate was *40A*, with a relative rate: 0.67 (95% CI [0.39, 1.14]) which overlaps the baseline rate, and with the confidence interval of the fastest strain (Fig. 2b). We thus conclude that while differences could exist between the sporulation rates, the results are not significant. There was considerable variation between the strains in the average time to producing their first immature chasmothecia, the difference in means being 3.83 days between fastest and slowest strains. The two fastest strains (*4000* and *876–1*) had relative rates of 3.25 and 2.49, with confidence intervals that did not overlap the slowest strain, *i1* this was 0.74. This is seen both in panels c and d of Fig. 2, indicating that the significant difference exists regardless of whether we analyse the time from sporulation to chasmothecia or the time from the beginning of the experiment to the timing of the chasmothecia. Further summary statistics for all traits analysed are given in Table S10 in Additional file 1 Chapter 6.2.

### Infection abundance measures

Pairwise model comparisons (Tables S11 and S12 in Additional file 1 Chapter 7.1) confirmed also that both strain identity and experiment identity were significant predictors for predicting pathogen infection abundances: i.e. Bevan scale at day 15 and the final chasmothecia category. Analyses on the infection abundance revealed a significantly lower infection abundance for the strain i1 at day 15, and on the other hand significantly more chasmothecia produced for strains 876–1 and 747–4 by the end of the follow-up. The analyses also strongly suggested that slow growth coincides with lower infection abundance, as the estimated effects of infection timings (first sporulation or first chasmothecia) on abundance were significant and negative (Tables S13 and S14 in the Additional file 1 Chapter 7.2).

### Correlations between life-history traits

When studying correlations between the life-history traits, we found that the speed of germination was significantly positively correlated with the timing of first sporulation, first immature and mature chasmothecia and the time it takes from sporulation to chasmothecia (Table S15 Chapter 8 Additional file 1). Moreover, the timing of first sporulation was significantly correlated with timing of first chasmothecia (Fig. 2e). We also found that the timing of first germination, sporulation, first immature and mature chasmothecia and time from sporulation to first chasmothecia all significantly correlated both with the Bevan scale at day 15 and the final chasmothecia category, so that faster developmental rate was associated with more abundant infection or chasmothecia (Fig. 2f, Table S16 Chapter 8 Additional file 1). Finally, we find that the timings of pathogen and hyperparasite infection stages were also for most of the cases positively and significantly correlated, meaning that fast growth of pathogen induces fast growth of the hyperparasite as well (Table S17 in Chapter 8 of the Additional file 1). Thus to conclude, the rates at which different strains accomplish different life-history stages are to a large extent positively correlated, also with the rates of the hyperparasite, and faster developmental rates correspond to more abundant spore production.

### Sensitivity to the hyperparasite

**Fig. 3:**
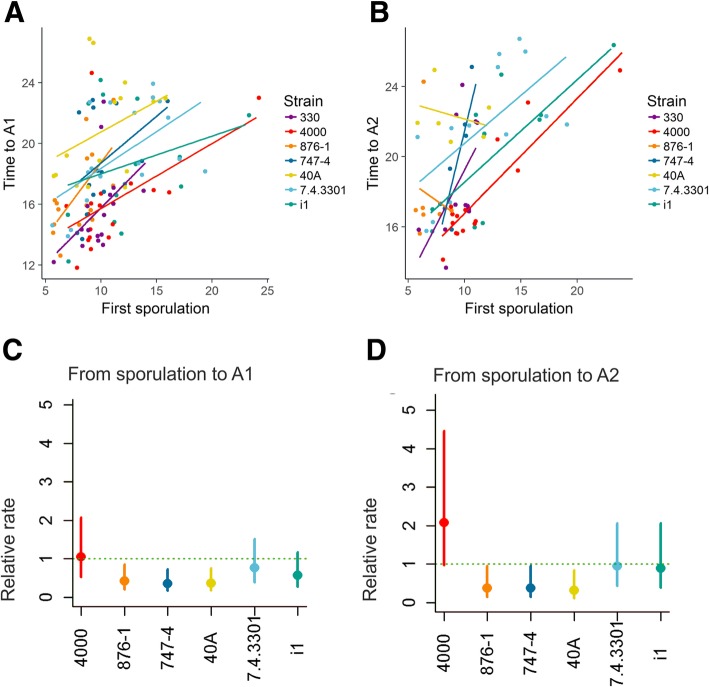
Correlations between pathogen and hyperparasite life-history traits. Panels **a-b** show the correlation in timings (in days) between the powdery mildew and hyperparasite life-history stages (time to reach A1 and A2). Panels **c-d** show the corresponding relative rates

All pairwise model comparisons identified strain as a significant predictor for the different measured characteristics of hyperparasite infection progression (Table S18 in Additional file 1). Taking into account the pathogen infection state at the time of hyperparasite infection, on the other hand, was only significant when predicting the times to A1 and A2, but not when times from sporulation to A1 and A2 were considered (Table S19 in Additional file 1). There were significant differences between the strains in the sensitivity to the hyperparasite, as suggested by the between-strain difference in the timings of hyperparasite infectivity stages (for example between *4000* and *40A*, Fig. 3a-b, Table S21 Chapter 9 of the Additional file 1). However, these differences were somewhat mitigated when the pathogen infection status at the time of inoculation of the hyperparasite was taken into account (Table S22 Chapter 9 of the Additional file 1). Yet regardless of the modelling assumption, the fastest growing strain 4000 is associated with faster development from mildew sporulation to state A2 (Tables S21 and S22 in the Additional file 1). Also, even when the pathogen infection status at the time of hyperparasite inoculation was accounted for when modelling hyperparasite infection development, the pairwise model comparisons suggested the pathogen strain as a significant predictor (Table S20 in the Additional file 1). However, the analyses on trait correlations revealed that these processes are strongly correlated with the growth rates of the mildew. Pairwise analyses of the timing of powdery mildew and hyperparasite infection stages revealed that earlier timing of hyperparasite reaching infection stage A1 was significantly associated with earlier timing of germination, sporulation and shorter time from germination to sporulation of the powdery mildew (Fig. 3c, Table S17 in Chapter 8 of the Additional file 1), and similar correlations were found when modelling the timing of A2. The time from sporulation to A2 was also positively associated with timing of sporulation and chasmothecia. Jointly our results suggest that faster mildew growth coincides with faster hyperparasite growth and that this correlation is evident at both within and between strain levels.

### Differences in the estimated pathogen traits and link to metapopulation prevalence

To understand how variation in life-histories impact on realized fitness, we assessed whether any of the life-history traits was associated with prevalence in the natural pathogen metapopulation. In 2015, pathogens with corresponding MLG-profiles to the strains studied here were found thoughout the metapopulation, as seen from Figure S2 in Chapter 3 of the Additional file 1 Y. The most prevalent among the strains, used in our laboratory study, *i1*, was found at 62 locations, while strains *40A* and *747–4* were only found at 4 locations Figure S2 in Chapter 3 of the Additional file 1. We tested correlations between the trait means of the strains and their observed prevalence. While such comparisons are restricted by the small number of considered strains (*N* = 7), the overall conclusion of these analyses, as well as from visual inspections was, was that none of the measured life-history traits were directly associated with prevalence in the wild (Figure S5 in Chapter 10 of the Additional file 1). Interestingly, the most prevalent strain in nature, *i1*, did not rank the highest in any of our laboratory measured fitness traits.

## Discussion

Understanding the mechanisms by which diversity is generated and maintained in pathogen populations has been a long-standing challenge in disease biology [23, 53]. Resolving this is non-trivial given that diversity of pathogen populations underlies risks of infection and disease emergence. Here, we have documented both asexual and sexual stages as well as sensitivity to a hyperparasite of an obligate fungal pathogen. Although we measured significant variation among *P. plantaginis* strains in their life-history traits that constitute the infection cycle, we did not find any evidence for trade-offs among pathogen development stages. However, we show that fast growth comes at a cost of being more sensitive to the hyperparasite. This finding highlights that pathogen coevolution with their host may be constrained by the antagonistic interactions with other species. Further, we find that fitness differences observed between pathogen strains in vitro did not translate into corresponding in vivo differences in abundance, which suggests that there may be multiple life-history strategies for being common at the metapopulation scale.

Our inoculation study revealed that there are strains which undergo the successive life-history stages faster, and the same strains are observed to produce more transmission propagules (conidia) as well as more sexual resting spores (chasmothecia) at the end of an infection. Likewise, we find that other strains are consistently slower at completing their life-cycle, leading to lower infection load on their host. Although negative correlations between pathogen life-history stages have been frequently found [23], there is also evidence of positive correlations in other pathogens [28, 34]. Investigations of pathogen life-history typically do not include the sexual stage [23], likely due to the empirical challenges associated with quantifying this stage [7]. Here, we did not find any evidence of a trade-off between asexual and sexual stages. In contrast, strains that produced a high number of clonal conidia also yielded more chasmothecia. However, as our study only quantified chasmothecia produced via haploid selfing, we cannot rule out the possibility that there is a cost between the asexual stage and sexual reproduction via outcrossing. Investment in asexual and sexual stages in pathogens is still largely unexplored, and hence offers an exciting avenue of future research with the potential to reveal how diversity is maintained in pathogen life-history stages.

In our experiments the main trade-off we found was that fast pathogen growth comes at the cost of faster growth of the hyperparasite, *Ampelomyces.* This is in line with a previous result showing that *P. plantaginis* strains differ significantly in their sensitivity to *Ampelomyces* [30], but to our knowledge, this is the first report showing that pathogen life-history traits may be in trade-off with sensitivity to a hyperparasite. Field observations in this pathosystem have demonstrated that hyperparasitism has severe fitness consequences for this pathogen by significantly reducing the probability of successful overwintering [49], and so for some pathogen strains it may be adaptive to employ a life-history strategy that mitigates hyperparasitism risk. The correlations between life history traits and susceptibility to the *Ampelomyces* hyperparasite are driven by both within- and between strain variation. The within-strain phenotypic plasticity could in itself be selected for if strains can optimize their phenotypes to current conditions [17]. Variation among the strains in these correlations may be a powerful mechanisms for promoting variation in these traits [17]. More research is needed in this system to assess in detail if infection with the hyperparasite can offset the differences in growth rate and fitness we observe between strains. It would also be interesting to verify the current results across a broader range of genotypic variation of the host plant, its pathogen and the hyperparasite. Strong genotype-by-genotype interactions could generate spatio-temporal variation in life-history allocations in this pathosystem.

## Conclusions

We conclude that the studied powdery mildew strains exhibit both within- and between strain variation in their life-history traits related to the different reproduction stages. We also find that life-history traits are mostly positively correlated, so that fast growth at one stage predicts similar growth at the subsequent stages. This leads to higher reproductive fitness of the initially fast strains. However, our results suggest that variation in the risk of hyperparasitism across the natural pathogen metapopulation coupled with some environmental and stochastic factors may act to maintain variation in life-history strategies among powdery mildew strains. Genotype-by-environment interactions may change ranking of strains across environmental gradients and even alter the shape of trade-offs. Indeed, the outcome of host-pathogen interactions is often mediated by both biotic and abiotic variation of the environment [4, 39, 51, 52]. This brings us to the conclusion that while studying pathogen strains in laboratory conditions sheds immediate light on the life-history traits and their constraints, more work is needed on how these traits are modulated by the environment, how they are related to the data collected from the natural conditions, and finally, how the two kinds information can be utilized to make robust predictions regarding disease dynamics in nature.

## Additional file

Additional file 1: **Figure S1.** Summary of the experimental design. **Table S1.** The number of of inoculations per strain in each experiment block. **Table S2.** The total numbers of inoculations that germinated for each strain. **Table S3.** The total numbers of inoculations that resulted in immature and mature chasmothecia. **Table S4.** The number of Ampelomyces hyperparasite inoculations that reached state A1 for each powdery mildew strain. **Table S5.** The number of Ampelomyces hyperparasite inoculations that reached hyperparasite state A2 for each powdery mildew strain. **Table S6.** Typer4 Parameters for genotyping calling. **Figure S2.** Panel (A) displays the locations in which the studied strains were found in 2015. Majority (395) of all the strains found that year were only found in one location. Panel (B) shows the frequency distribution for the number of occupied locations for all the strains, for the SNP panel of 19 SNPs together with the locus with contig ID c6190. The study strains are shown in colors, and the amounts of colonized locations for each strain are shown in parenthesis. The shape_les used for generating the maps in panel A were downloaded from Stanford digital repository (https://purl.stanford.edu/np067sb6776). **Figure S3.** Powdery mildew growth was scored using Bevan’s scale (adapted from [7]), ranging from 0 to 4 (0: no mycelium, 1: mycelium only, 2: mycelium and sparse sporulation visible only under a dissecting microscope, 3:abundant sporulation and lesion size < 0.5 cm2, 4: abundant sporulation and lesion size > 0.5 cm2). **Figure S4.** Ampelomyces infection was scored with a modi_ed version of the scale reported in [16]: A0: no pycnidia observed, A1: 1–20 pycnidia in each Ampelomyces cluster appearing and A2: 20–50 pycnidia in each powdery mildew lesion or between 30 and 50% of powdery mildew covered. This scale can re_ect either a set number of Ampelomyces pycnidia or an estimate of pycnidia coverage of the powdery mildew lesion. Hence, the scale controls for the di_erent amounts of powdery mildew tissue available for the hyperparasite to infect, i.e. small powdery mildew lesions can still support hyperparasite infection state A2 even if there is not enough tissue to produce abundant pycnidia. **Table S7.** The means and standard deviations of the measured life-history traits. Summary statistics for the timings of life-history events are computed only for inoculations for which the event actually occurred. The largest average value in each row is indicated in red, and the smallest in blue. **Table S8.** The results from pairwise model comparisons, for the survival models where the model with only strain id and the same model with both the experiment id and strain id as predictors are contrasted using anova. The presented *p*-value corresponds to the evidence in favor of the more rich model (Model 2). **Table S9.** The results from pairwise model comparisons, for the survival models where the model with only experiment id and the same model with both the experiment id and strain id as predictors are contrasted using anova. The presented *p*-value corresponds to the evidence in favor of the more rich model (Model 2). **Table S10.** The estimated relative rates and their 95% con_dence intervals for the di_erent studied infection event times, together with the associated test statistic for rejecting the null hypothesis of the corresponding factor having no e_ect on the rate of the event. Signi_cant deviations are shown in bold. **Table S11.** The results from pairwise model comparisons, for the ordinal regression models where the model with only experiment id and the same model with both the experiment id and strain id as predictors are contrasted using anova. The presented p-value corresponds to the evidence in favor of the more rich model (Model 2). **Table S12.** The results from pairwise model comparisons, for the ordinal regression models where the model with only strain id and the same model with both the experiment id and strain id as predictors are contrasted using anova. The presented p-value corresponds to the evidence in favor of the more rich model (Model 2). **Table S13.** Estimated e_ects for the ordinal regression model for the Bevan scale at day 15. Signi_cant deviations are shown in bold. **Table S14.** Estimated e_ects for the ordinal regression model for the (immature) chasmothecia category by the end of the follow-up. **Table S15.** The estimated relative rates (exponentials of the estimated coe_cients) for using the columns as predictor when predicting the event times indicated by the rows. The statistical signi_cancy of the estimated e_ect is shown in parenthesis and the signi_cant e_ects are shown in bold. With NA’s we have omitted the pairs of events occurring in wrong order, as they lead to non-intuitive modelling, as well as. **Table S16.** The estimated e_ects of event timings (columns) as predictor when predicting the abundance measureds indicated by the rows. The statistical signi_cancy of the estimated e_ect is shown in parenthesis and the signi_cant e_ects are shown in bold. **Table S17.** The estimated relative rates (exponentials of the estimated coe_cients) for using event times in the columns as predictor for the hyperparasite infection event times, indicated by the rows. The statistical signi_cancy of the estimated e_ect is shown in parenthesis and the signi_cant e_ects are shown in bold. **Table S18.** The results from pairwise model comparisons, for the survival models presented in where a survival model without any predictors and a model with the strain as a predictor are contrasted using anova. The presented p-value corresponds to the evidence in favor of the more rich model (Model 2). **Table S19.** The results from pairwise model comparisons, for survival models where a survival model with strain id as a predictor and a model with the strain and pathogen infection status at day 8 as a predictor are contrasted using anova. The presented p-value corresponds to the evidence in favor of the more rich model (Model 2). **Table S20.** The results from pairwise model comparisons, for the survival models where a survival model with pathogen infection status at day 8 as a predictor and a model with the strain id and pathogen infection status at day 8 as a predictor are contrasted using anova. The presented p-value corresponds to the evidence in favor of the more rich model (Model2). **Table S21.** The estimated relative rates and their 95% con_dence intervals for the di_erent studied hyperparasite infection event times, together with the associated test statistic for rejecting the null hypothesis of the corresponding factor having no e_ect on the rate of the event. Signi_cant deviations are shown in bold. **Table S22.** The estimated relative rates and their 95% con_dence intervals for the di_erent studied hyperparasite infection event times, where in the model the pathogen infection stage at day 8 was accounted for, together with the associated test statistic for rejecting the null hypothesis of the corresponding factor having no e_ect on the rate of the event. Signi_cant deviations are shown in bold. **Figure S5.** Panel A shows the frequency distribution for the number of occupied locations for all the observed strains in 2015. The majority of observed strains (395) in that year were only found in a single location, but 3 strains were found in > 25 discrete locations. Strains used to study life-history variation are shown in color. In panel B the prevalence of strains across the metapopulation is correlated with the mean _tness traits, and the corresponding *p*-values for the _tted rank correlations are shown in upright. (PDF 1859 kb)

## Data Availability

Data available from the Dryad Digital Repository: 10.5061/dryad.56b7s48

## Abbreviations

DPI: Days post inoculation
MLG: Multilocus genotype
SNP: Single nucleotide polymorphism

## References

[1] Abo-Foul S, Raskin V, Sztejnberg A, Marder J (1996). Disruption of chlorophyll organization and function in powdery mildew-diseased cucumber leaves and its control by the hyperparasite Ampelomyces quisqualis. Phytopathology.

[2] Agrios GN (2005). Plant pathology.

[3] Andersen SB, Ferrari M, Evans HC, Elliot SL, Boomsma JJ, Hughes DP (2012). Disease dynamics in a specialized parasite of ant societies. PLoS One.

[4] Attisano A, Moore A, Moore P (2012). Reproduction-longevity trade-offs reflect diet, not adaptation. J Evol Biol.

[5] Auld SKJR, Tinsley MC (2015). The evolutionary ecology of complex lifecycle parasites: linking phenomena with mechanisms. Heredity.

[6] Bevan JR, Crute IR, Clarke DD (1993). Diversity and variation in expression of resistance to *Erysiphe fischeri* in *Senecio vulgaris*. Plant Pathol.

[7] Billiard S, López-Villavicencio M, Hood M, Giraud T (2012). Sex, outcrossing and mating types: unsolved questions in fungi and beyond. J Evol Biol.

[8] Blanchong JA, Robinson SJ, Samuel MD, Foster JT (2016). Application of genetics and genomics to wildlife epidemiology. J Wildl Manag.

[9] Bryner SF, Rigling D (2010). Temperature-dependent genotype-by-genotype interaction between a pathogenic fungus and its hyperparasitic virus. Am Nat.

[10] Choi GH, Nuss DL (1992). Hypovirulence of chestnut blight fungus conferred by an infectious viral cDNA. Science.

[11] Christensen RHB (2010). Ordinal—regression models for ordinal data.

[12] Cox DR (1972). Regression models and life-tables. J R Stat Soc Ser B Methodol.

[13] Falk SP, Gadoury DM, Pearson RC, Seem RC (1995). Partial control of grape powdery mildew by the mycoparasite *Ampelomyces quisqualis*. Plant Dis J.

[14] Fraile A, Pagan I, Anastasio G, Saez E, Garcia-Arenal F (2011). Rapid genetic diversification and high fitness penalties associated with pathogenicity evolution in a plant virus. Mol Biol Evol.

[15] Friesen TL, Stukenbrock EH, Liu Z, Meinhardt S, Ling H, Faris JD (2006). Emergence of a new disease as a result of interspecific virulence gene transfer. Nat Genet.

[16] Giraud T, Enjalbert J, Fournier E, Delmotte F, Dutech C (2008). Population genetics of fungal diseases of plants. Parasite.

[17] Hendry AP (2016). Key questions on the role of phenotypic plasticity in eco-evolutionary dynamics. J Hered.

[18] Holt RD, Hochberg ME (1998). The coexistence of competing parasites. Part II—Hyperparasitism and food chain dynamics. J Theor Biol.

[19] Jousimo J, Tack AJ, Ovaskainen O, Mononen T, Susi H, Tollenaere C (2014). Ecological and evolutionary effects of fragmentation on infectious disease dynamics. Science.

[20] Koskella B (2013). Phage-mediated selection on microbiota of a long-lived host. Curr Biol.

[21] Laine A-L (2004). A powdery mildew infection on a shared host plant affects the dynamics of the Glanville fritillary butterfly populations. Oikos.

[22] Laine A-L, Hanski I (2006). Large-scale spatial dynamics of a specialist plant pathogen in a fragmented landscape. J Ecol.

[23] Laine AL, Barrès B (2013). Epidemiological and evolutionary consequences of life-history trade-offs in pathogens. Plant Pathol.

[24] Mundt C (2002). Use of multiline cultivars and cultivar mixtures for disease management. Annu Rev Phytopathol.

[25] Nemri A, Barrett LG, Laine A-L, Burdon JJ, Thrall PH (2012). Population processes at multiple spatial scales maintain diversity and adaptation in the Linum marginale-Melampsora lini association. PLoS One.

[26] Nicot P, Bardin M, Dik A (2002). Basic methods for epidemiological studies of powdery mildews: culture and preservation of isolates, production and delivery of inoculum, and disease assessment. The Powdery Mildews: a comprehensive treatise.

[27] Nieminen M, Siljander M, Hanski I (2004). Structure and dynamics of Melitaea cinxia metapopulations. On the wings of checkerspots: a model system for population biology.

[28] Pariaud B, Berg F, Bosch F, Powers SJ, Kaltz O, Lannou C (2013). Shared influence of pathogen and host genetics on a trade-off between latent period and spore production capacity in the wheat pathogen, Puccinia triticina. Evol Appl.

[29] Parratt SR, Laine A-L (2016). The role of hyperparasitism in microbial pathogen ecology and evolution. ISME J.

[30] Parratt SR, Barrès B, Penczykowski RM, Laine AL. Local adaptation at higher trophic levels: contrasting hyperparasite–pathogen infection dynamics in the field and laboratory. Mol Ecol. 2017;26:1964-79. 10.1111/mec.13928. 10.1111/mec.13928 PMC5412677 27859910

[31] Parratt SR, Laine A-L (2018). Pathogen dynamics under both bottom-up host resistance and top-down hyperparasite attack. J Appl Ecol.

[32] Rasmussen AL, Katze MG (2016). Genomic signatures of emerging viruses: a new era of systems epidemiology. Cell Host Microbe.

[33] Romero D, Rivera ME, Cazorla FM, De Vicente A, Perez-Garcia A (2003). Effect of mycoparasitic fungi on the development of Sphaerotheca fusca in melon leaves. Mycol Res.

[34] Salvaudon L, Héraudet V, Shykoff JA, Koella J (2005). Parasite-host fitness trade-offs change with parasite identity: genotype-specific interactions in a plant-pathogen system. Evolution.

[35] Schmid Hempel P (2011). Evolutionary parasitologythe integrated study of infections, immunology, ecology, and genetics.

[36] Springer JC, Baines ALD, Fulbright DW, Chansler MT, Jarosz AM (2013). Hyperparasites influence population structure of the chestnut blight pathogen, *Cryphonectria parasitica*. Phytopathology.

[37] Stearns SC (1988). Citation classic - the evolution of life-history traits - a critique of the theory and a review of the data. Curr Contents/Agric Biol Environ Sci.

[38] Stearns SC (1989). Trade-offs in life-history evolution. Funct Ecol.

[39] Susi H, Laine AL (2013). Pathogen life-history trade-offs revealed in allopatry. Evolution.

[40] Susi H, Laine AL (2015). The effectiveness and costs of pathogen resistance strategies in a perennial plant. J Ecol.

[41] Szentiványi O, Kiss L (2003). Overwintering of *Ampelomyces* mycoparasites on apple trees and other plants infected with powdery mildews. Plant Pathol.

[42] Tack AJ, Laine AL (2014). Ecological and evolutionary implications of spatial heterogeneity during the off-season for a wild plant pathogen. New Phytol.

[43] Tack AJ, Thrall PH, Barrett LG, Burdon JJ, Laine AL (2012). Variation in infectivity and aggressiveness in space and time in wild host–pathogen systems: causes and consequences. J Evol Biol.

[44] Talbot NJ (2015). Taming a wild beast: developing molecular tools and new methods to understand the biology of *Zymoseptoria tritici*. Fungal Genet Biol.

[45] Taylor DR, Jarosz AM, Fulbright DW, Lenski RE (1998). The acquisition of hypovirulence in host-pathogen systems with three trophic levels. Am Nat.

[46] Therneau T (2015). A Package for Survival Analysis in S. version 2.38.

[47] Thrall PH, Burdon JJ (2003). Evolution of virulence in a plant host-pathogen metapopulation. Science.

[48] Tollenaere C, Laine AL (2013). Investigating the production of sexual resting structures in a plant pathogen reveals unexpected self-fertility and genotype-by-environment effects. J Evol Biol.

[49] Tollenaere C, Pernechele B, Mäkinen H, Parratt S, Németh M, Kovács G (2014). A hyperparasite affects the population dynamics of a wild plant pathogen. Mol Ecol.

[50] Tollenaere C, Susi H, Nokso-Koivisto J, Koskinen P, Tack A, Auvinen P (2012). SNP design from 454 sequencing of *Podosphaera plantaginis* transcriptome reveals a genetically diverse pathogen metapopulation with high levels of mixed-genotype infection. PLoS One.

[51] Vale PF, Wilson AJ, Best A, Boots M, Little TJ (2011). Epidemiological, evolutionary and co-evolutionary implications of context-dependent parasitism. Am Nat.

[52] Wolinska J, King KC (2009). Environment can alter selection in host–parasite interactions. Trends Parasitol.

[53] Woodhams DC, Alford RA, Briggs CJ, Johnson M, Rollins-Smith LA (2008). Life-history trade-offs influence disease in changing climates: strategies of an amphibian pathogen. Ecology.

[54] Yau S, Lauro FM, DeMaere MZ, Brown MV, Thomas T, Raftery MJ (2011). Virophage control of antarctic algal host–virus dynamics. Proc Natl Acad Sci.

