# Supplementary material for: Variation and correlations between sexual, asexual and natural enemy resistance life-history traits in a natural plant pathogen population
Source: BMC Evol Biol. 2019 Jul 12;19:142. doi: 10.1186/s12862-019-1468-2 (PMC6624897; doi:10.1186/s12862-019-1468-2)
Supplement: Supplementary file 1 — Figure S1. Summary of the experimental design. Table S1. The number of of inoculations per strain in each experiment block. Table S2. The total numbers of inoculations that germinated for each strain. Table S3. The total numbers of inoculations that resulted in immature and mature chasmothecia. Table S4. The number of Ampelomyces hyperparasite inoculations that reached state A1 for each powdery mildew strain. Table S5. The number of Ampelomyces hyperparasite inoculations that reached hyperparasite state A2 for each powdery mildew strain. Table S6. Typer4 Parameters for genotyping calling. Figure S2. Panel (A) displays the locations in which the studied strains were found in 2015. Majority (395) of all the strains found that year were only found in one location. Panel (B) shows the frequency distribution for the number of occupied locations for all the strains, for the SNP panel of 19 SNPs together with the locus with contig ID c6190. The study strains are shown in colors, and the amounts of colonized locations for each strain are shown in parenthesis. The shape_les used for generating the maps in panel A were downloaded from Stanford digital repository (https://purl.stanford.edu/np067sb6776). Figure S3. Powdery mildew growth was scored using Bevan’s scale (adapted from [7]), ranging from 0 to 4 (0: no mycelium, 1: mycelium only, 2: mycelium and sparse sporulation visible only under a dissecting microscope, 3:abundant sporulation and lesion size < 0.5 cm2, 4: abundant sporulation and lesion size > 0.5 cm2). Figure S4. Ampelomyces infection was scored with a modi_ed version of the scale reported in [16]: A0: no pycnidia observed, A1: 1–20 pycnidia in each Ampelomyces cluster appearing and A2: 20–50 pycnidia in each powdery mildew lesion or between 30 and 50% of powdery mildew covered. This scale can re_ect either a set number of Ampelomyces pycnidia or an estimate of pycnidia coverage of the powdery mildew lesion. Hence, the scale controls for the di_erent amounts of [file 12862_2019_1468_MOESM1_ESM.pdf]

Supplementary materials for the article entitled *Variation and correlations between sexual, asexual, and natural enemy resistance life-history traits in natural plant pathogen population*

Elina Numminen *[1], Elise Vaumourin[1], Lucie Poulin[2], Steven Parratt[3], and Anna-Liisa Laine[1,4]

[1]*Research Centre for Ecological Change, University of Helsinki, Faculty of biological and environmental sciences, FIN-00014, University of Helsinki, Finland*
[2]*Universite de Nantes, Faculte des Sciences et des Techniques, Laboratoire de Biologie et de Pathologie Vegetales (LBPV), EA 1157, SFR 4207 QUASAV, 2, rue de la Houssiniere, BP 92 208, F-44322 Nantes Cedex 3, France*
[3]*University of Liverpool, Institute of Integrative biology, Liverpool L69 3BX, United Kingdom*
[4]*Department of Evolutionary Biology and Environmental Studies University of Zurich, Winterthurerstrasse 190 CH-8057 Zurich, Switzerland*

*Corresponding author. elina.numminen@helsinki.fi, Viikinkaari 1, PO Box 65, FI-00014 University of Helsinki, Finland.

Contents

1 Experimental setup **3**
- 1.1 Inoculation experimental design . . . 3
- 1.2 Success rate of the inoculations . . . 4
- 1.3 Success rate of the *Ampelomyces* infection . . . 4

2 Genotyping **5**

3 Metapopulation strain diversity **6**

4 Quantifying infection severity in the pathogen and the hyperparasite **7**
- 4.1 Pathogen infection stages . . . 7
- 4.2 Hyperparasite infection stages . . . 8

5 Summary statistics of the life-history traits **9**

6 Survival model fits for the pathogen **10**
- 6.1 Pairwise model comparisons for the timings of powdery mildew infection stages . . . 10
- 6.2 Timings of pathogen infection stages . . . 11

7 Abundance models **13**
- 7.1 Pairwise model comparisons for abundance models . . . 13
- 7.2 Bevan scale at day 15 . . . 14
- 7.3 Final immature chasmothecia category . . . 14

8 Life-history trait correlations **15**

9 Survival model fits for the hyperparasite **16**
- 9.1 Pairwise model comparisons for the timings of hyperparasite infection stages . . . 16
- 9.2 Timings of hyperparasite infection stages . . . 17
  - 9.2.1 Without the pathogen infection status as a predictor . . . 17
  - 9.2.2 With the pathogen infection status as a predictor . . . 18

10 Fitness traits and metapopulation prevalence **19**

11 Bibliography **20**

1 Experimental setup

1.1 Inoculation experimental design

The experiments to characterise the life-history traits (LHT) of the seven studied powdery mildew strains (time to germination, time to sporulation, Bevan scale measures, time of appearance and maturation of the chasmothecia, number of immature and mature chasmothecia) were conducted in three blocks, conducted between December 2015 and May 2016. The experiment blocks varied with respect to the exact experimental monitoring duration (in days) and the minimum number of inoculations per strain within them (in leaves), as shown in Figure 1. Table 1 shows the number of inoculations with each strain within each experiment block.

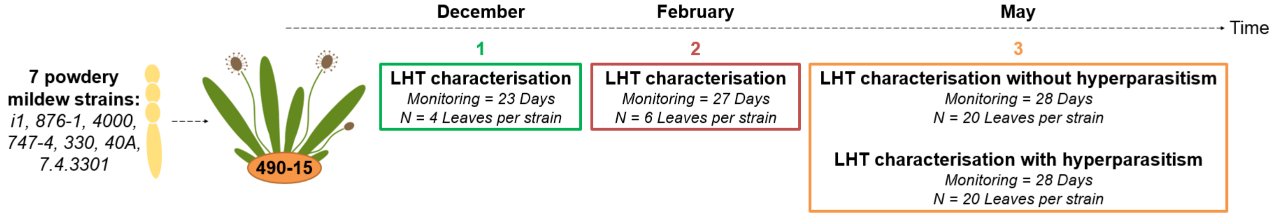

Figure 1: Summary of the experimental design

Experiment	330	4000	876-1	747-4	40A	7.4.3301	i1
1	4	4	4	4	4	4	4
2	6	7	9	6	8	8	6
3 without hyperparasitism	20	20	20	20	20	20	20
3 with hyperparasitism	20	20	20	20	20	20	20

Table 1: The number of of inoculations per strain in each experiment block

1.2 Success rate of the inoculations

The inoculations had a high success rate as only 15 of them never germinated. All these inoculations were from the experiment block 3, which also contained most of the data. Table 2 shows how the unsuccessful inoculations were distributed across the strains. All the inoculations that germinated also eventually sporulated.

Table 2: The total numbers of inoculations that germinated for each strain.

	Did not germinate	Germinated
330	5	47
4000	1	50
876-1	0	53
747-4	1	49
40A	6	48
7.4.3301	0	52
i1	2	58

There was considerable variation between the strains in the number of inoculations that reached the final life-history stages of produced chasmothecia (either immature or mature), as seen in Table 3. As especially particularly low number of inoculations lead into mature chasmothecia for some strains (*i1* and *330*), we suspect that the time-span of the follow up was not long enough for these strains having the time to mature. For this reason we focused our analyses mostly on the timing of the first chasmothecia.

Table 3: The total numbers of inoculations that resulted in immature and mature chasmothecia

	chasmothecia	mature chasmothecia
330	35	16
4000	45	36
876-1	40	38
747-4	43	29
40A	38	26
7.4.3301	37	20
i1	28	9

1.3 Success rate of the *Ampelomyces* infection

All the hyperparasite inoculations were conducted in the experiment block 3. There was a considerable variation in the hyperparasite infection success, as seen from Tables 4 and 5.

Table 4: The number of *Ampelomyces* hyperparasite inoculations that reached state A1 for each powdery mildew strain.

	330	4000	876-1	747-4	40A	7.4.3301	i1
No Event	2	3	5	6	3	2	6
Event	16	17	15	14	15	18	14

Table 5: The number of *Ampelomyces* hyperparasite inoculations that reached hyperparasite state A2 for each powdery mildew strain.

	330	4000	876-1	747-4	40A	7.4.3301	i1
No Event	6	5	13	13	12	7	10
Event	12	15	7	7	6	13	10

2 Genotyping

In September 2015 all pathogens from all infected host populations were sampled for genotyping by collecting infected leaves into individual paper envelopes and drying them prior to DNA extraction. Between 1 and 10 samples – always from different plants - were collected from each population, with the number of samples adjusted to local pathogen population size. In the laboratory powdery mildew lesions were removed by a scalpel into individual wells of 96 well plates which were further processed for DNA extraction at BI (Institute of Biotechnology, University of Helsinki) using the E.Z.N.A. Plant DNA Kit according to the manufacturer's protocols (Omega Bio-tek, Inc.). DNA samples were genotyped with a 19 SNP genotyping panel that was developed in our lab (Tolleanaere et al. 2014) using the Sequenom MassARRAY iPLEX platform. The identity of each SNP was called using the MassARRAY Typer 4 software (Sequenom, San Diego, CA) using the parameters detailed in the Table 6, see also (Tollenaere et al. 2014). Here the high and low mass refer to thresholds required for the software to make a call, heterozygosity refers toa threshold for distinguishing heterozygotes from homozygotes and magnitude corresponds to overall signal strength. As strains trains *4000* and *747-4* appeared similar in the 19 SNPs, we considered a full panel consisting of altogether 27 SNPs, finding that they differed in locus with contig id $c6190$.

Table 6: Typer4 Parameters for genotyping calling

SNP ID	contig ID	Low mass	High mass	Heterozygosity	Magnitude
harma c1217 640	c1217	1/18	1/18	1/25	6.5
harma c1336 788	c1336	1/18	1/18	1/25	8
harma c1421 219	c1421	1/18	1/18	1/25	6
harma c1421 455	c1421	1/18	1/18	1/25	6
harma c1720 2036	c1720	1/18	1/18	1/25	6
harma c1892 2119	c1892	1/18	1/18	1/25	6
harma c2493 601	c2493	1/18	1/18	1/25	6
harma c2804 701	c2804	1/18	1/18	1/25	10
harma c3117 1457	c3117	1/18	1/18	1/25	6
harma c3926 348	c3926	1/18	1/18	1/25	6
harma c3997 419	c3997	1/18	1/18	1/25	7
harma c4769 1106	c4769	1/18	1/18	1/25	6
harma c5096 985	c5096	1/18	1/18	1/25	6
harma c5876 431	c5876	1/18	1/18	1/25	6
harma rep c542 236	rep c542	1/18	1/18	1/25	6
harma rep c6068 457	rep c6068	1/18	1/18	1/25	6
harma rep c664 2300	rep c664	1/18	1/18	1/25	6
harma rep c707 1118	rep c707	1/18	1/18	1/25	6
harma rep c707 1234	rep c707	1/18	1/18	1/25	5

3 Metapopulation strain diversity

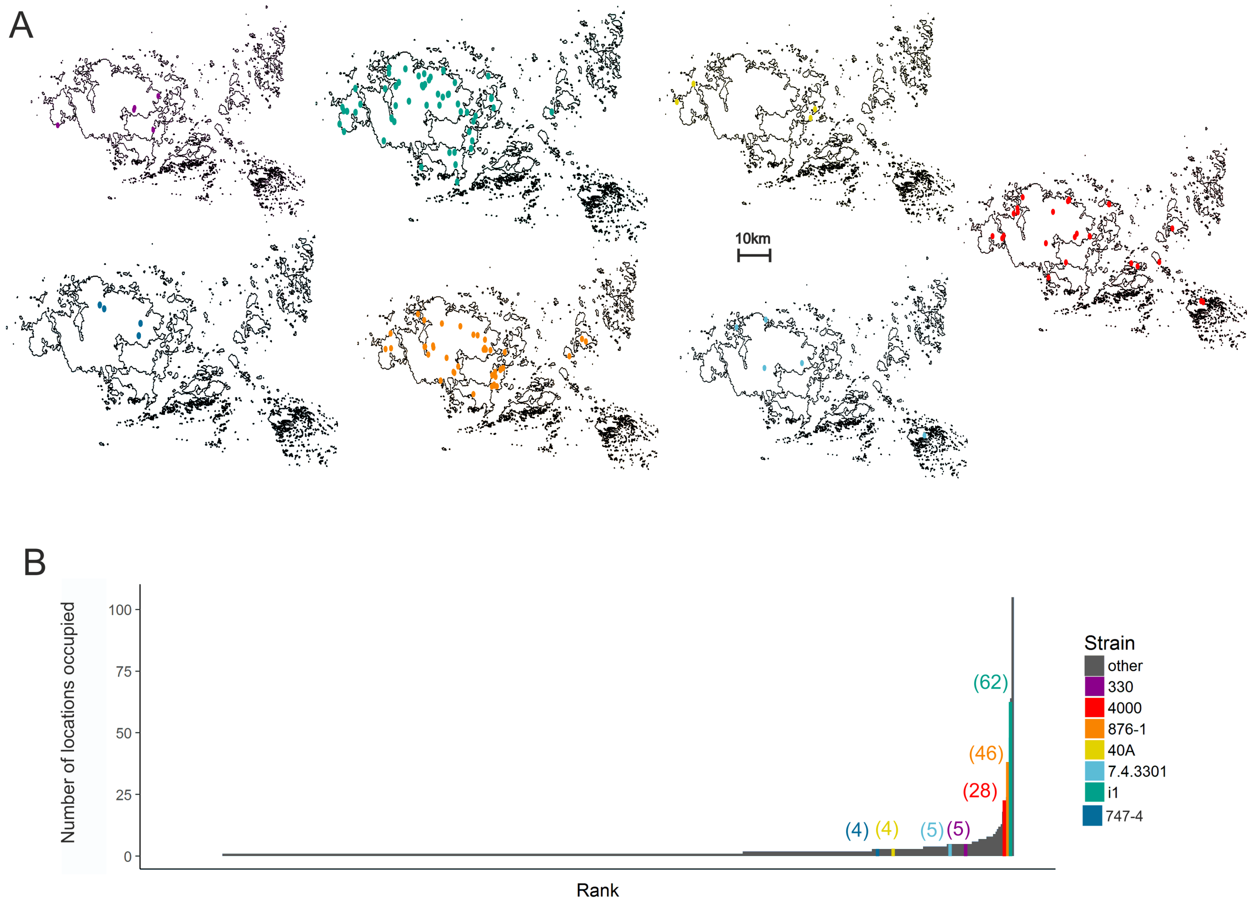

Figure 2: Panel (A) displays the locations in which the studied strains were found in 2015. Majority (395) of all the strains found that year were only found in one location. Panel (B) shows the frequency distribution for the number of occupied locations for all the strains, for the SNP panel of 19 SNPs together with the locus with contig ID *c*6190. The study strains are shown in colors, and the amounts of colonized locations for each strain are shown in parenthesis. The shapefiles used for generating the maps in panel A were downloaded from Stanford digital repository (https://purl.stanford.edu/np067sb6776).

4 Quantifying infection severity in the pathogen and the hyperparasite

4.1 Pathogen infection stages

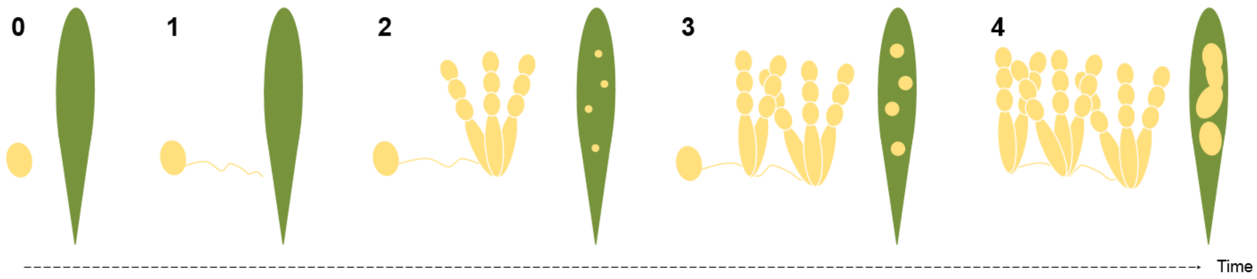

Figure 3: Powdery mildew growth was scored using Bevan's scale (adapted from (Bevan et al, 1993)), ranging from 0 to 4 (0: no mycelium, 1: mycelium only, 2: mycelium and sparse sporulation visible only under a dissecting microscope, 3: abundant sporulation and lesion size < $0.5cm^2$, 4: abundant sporulation and lesion size > $0.5cm^2$).

4.2 Hyperparasite infection stages

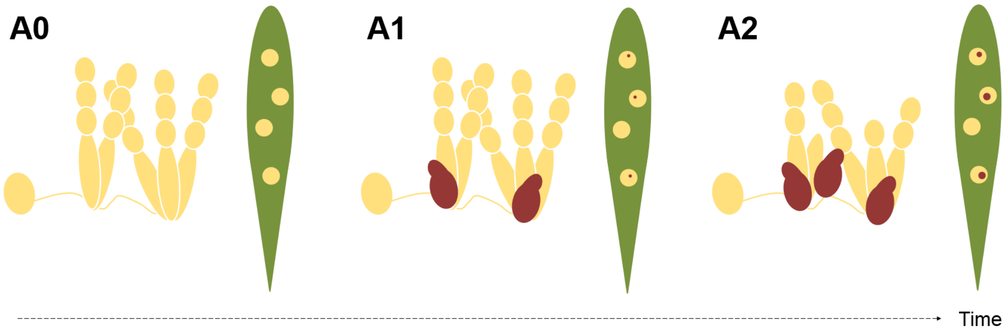

Figure 4: *Ampelomyces* infection was scored with a modified version of the scale reported in (Falk et al, 1995): A0: no pycnidia observed, A1: 1-20 pycnidia in each *Ampelomyces* cluster appearing and A2: 20-50 pycnidia in each powdery mildew lesion or between 30-50% of powdery mildew covered. This scale can reflect either a set number of *Ampelomyces* pycnidia or an estimate of pycnidia coverage of the powdery mildew lesion. Hence, the scale controls for the different amounts of powdery mildew tissue available for the hyperparasite to infect, i.e. small powdery mildew lesions can still support hyperparasite infection state A2 even if there is not enough tissue to produce abundant pycnidia.

5 Summary statistics of the life-history traits

Table 7: The means and standard deviations of the measured life-history traits. Summary statistics for the timings of life-history events are computed only for inoculations for which the event actually occurred. The largest average value in each row is indicated in red, and the smallest in blue.

		330	4000	876-1	747-4	40A	7.4.3301	i1
Bevan day 15	Mean	2.93	3.48	3.48	3.10	2.62	3.44	2.40
	SD	1.41	1.09	0.94	1.12	1.43	1.08	1.50
Final number of chasmothecia	Mean	0.70	1.71	1.94	1.20	0.94	1.06	0.60
	SD	0.65	1.07	1.48	0.89	0.95	0.95	0.81
First germination	Mean	3.19	2.67	3.18	3.17	3.32	2.53	2.89
	SD	1.86	1.35	1.89	2.21	1.96	0.98	1.45
First sporulation	Mean	9.41	8.57	9.36	11.00	10.50	9.41	10.14
	SD	1.95	2.27	2.51	4.06	2.76	2.38	4.03
First chasmothecia	Mean	18.35	14.52	15.23	16.44	16.91	16.96	17.56
	SD	4.64	3.63	4.01	3.28	3.62	4.69	4.85
From sporulation to chasmothecia	Mean	9.00	5.86	6.16	6.52	7.27	7.71	9.06
	SD	3.91	2.79	3.65	2.57	2.66	3.79	4.12
First mature chasmothecia	Mean	26.00	21.73	22.16	24.07	24.00	23.54	21.43
	SD	8.38	3.61	5.81	4.22	6.32	6.39	6.90
Time to A1	Mean	15.62	16.18	16.80	18.71	20.87	19.28	18.57
	SD	2.94	3.32	2.81	3.34	3.58	3.30	3.90
Time to A2	Mean	18.42	17.73	17.86	19.57	22.33	21.77	20.30
	SD	3.18	3.01	2.73	3.26	1.51	3.70	3.80
Time from sporulation to A1	Mean	5.81	5.12	8.80	8.71	10.60	7.39	6.29
	SD	2.69	3.67	2.14	3.15	3.81	3.38	4.95
Time from sporulation to A2	Mean	9.08	6.27	11.14	10.29	13.33	9.92	7.30
	SD	2.64	1.91	3.24	2.29	3.33	3.48	3.23
Time from A1 to A2	Mean	4.00	2.13	2.86	2.57	3.83	3.46	3.60
	SD	2.17	1.55	2.41	0.98	2.04	1.61	1.78
Ampelo infectivity day 15	Mean	0.50	0.35	0.10	0.10	0.00	0.10	0.10
	SD	0.62	0.59	0.31	0.31	0.00	0.31	0.31
Ampelo infectivity day 28	Mean	1.89	2.05	1.15	1.15	1.17	1.80	1.45
	SD	1.02	1.10	0.88	0.99	0.71	0.95	1.19

6 Survival model fits for the pathogen

6.1 Pairwise model comparisons for the timings of powdery mildew infection stages

Table 8: The results from pairwise model comparisons, for the survival models where the model with only strain id and the same model with both the experiment id and strain id as predictors are contrasted using anova. The presented p-value corresponds to the evidence in favor of the more rich model (Model 2).

Process	log-likelihood Model 1	log-likelihood Model 2	P(>|Chi|)
Time to germination	-898.04	-889.79	$\mathbf{0.01}$
Time to sporulation	-924.06	-914.34	$< \mathbf{10^{-2}}$
Time to chasmothecia	-816.15	-796.53	$< \mathbf{10^{-2}}$
From sporulation to chasmothecia	-790.41	-770.86	$< \mathbf{10^{-2}}$
Germination to sporulation	-899.16	-891.21	$\mathbf{0.014}$
Time to maturation	-547.48	-528.91	$< \mathbf{10^{-2}}$
From first chasmothecia to maturation	-488.20	-480.94	$\mathbf{0.02}$

Table 9: The results from pairwise model comparisons, for the survival models where the model with only experiment id and the same model with both the experiment id and strain id as predictors are contrasted using anova. The presented p-value corresponds to the evidence in favor of the more rich model (Model 2).

Process	log-likelihood Model 1	log-likelihood Model 2	P(>|Chi|)
Time to germination	-936	-890	< $< \mathbf{10^{-2}}$
Time to sporulation	-933	-914	$< \mathbf{10^{-2}}$
Time to chasmothecia	-816	-797	$< \mathbf{10^{-2}}$
From sporulation to chasmothecia	-778	-771	$< \mathbf{10^{-2}}$
Germination to sporulation	-893	-891	0.23
Time to maturation	-536	-529	$< \mathbf{10^{-2}}$
From first chasmothecia to maturation	-488	-481	$< \mathbf{10^{-2}}$

6.2 Timings of pathogen infection stages

Table 10: The estimated relative rates and their 95% confidence intervals for the different studied infection event times, together with the associated test statistic for rejecting the null hypothesis of the corresponding factor having no effect on the rate of the event. Significant deviations are shown in bold.

Event	Factor	exp.coef.	lower95.	upper95.	p-value
Time to germination	strain 4000	1.45	0.86	2.45	0.17
	strain 876-1	0.86	0.50	1.48	0.58
	strain 747-4	1.21	0.71	2.04	0.49
	strain 40A	0.69	0.40	1.18	0.17
	strain 7.4.3301	1.77	1.05	2.98	**0.03**
	strain i1	1.24	0.73	2.11	0.43
	experiment 2	0.43	0.26	0.71	$< \mathbf{10^{-2}}$
	experiment 3	0.11	0.06	0.17	$< \mathbf{10^{-2}}$
Time to sporulation	strain 4000	1.76	1.04	2.96	**0.03**
	strain 876-1	1.46	0.88	2.45	0.14
	strain 747-4	0.85	0.50	1.44	0.54
	strain 40A	0.67	0.39	1.14	0.14
	strain 7.4.3301	1.34	0.80	2.25	0.26
	strain i1	0.91	0.54	1.55	0.73
	experiment 2	0.71	0.44	1.14	0.16
	experiment 3	0.30	0.19	0.46	$< \mathbf{10^{-2}}$
Time to chasmothecia	strain 4000	3.25	1.85	5.71	$< \mathbf{10^{-2}}$
	strain 876-1	2.51	1.45	4.35	$< \mathbf{10^{-2}}$
	strain 747-4	1.28	0.73	2.27	0.39
	strain 40A	0.94	0.52	1.69	0.84
	strain 7.4.3301	1.03	0.58	1.82	0.93
	strain i1	0.74	0.40	1.38	0.35
	experiment 2	0.36	0.21	0.61	$< \mathbf{10^{-2}}$
	experiment 3	0.37	0.24	0.58	$< \mathbf{10^{-2}}$
From sporulation to chasmothecia	strain 4000	3.11	1.76	5.50	$< \mathbf{10^{-2}}$
	strain 876-1	2.16	1.25	3.72	**0.01**
	strain 747-4	1.59	0.90	2.82	0.11
	strain 40A	1.20	0.67	2.16	0.54
	strain 7.4.3301	0.87	0.49	1.54	0.63
	strain i1	0.64	0.34	1.19	0.15
	experiment 2	0.38	0.22	0.63	$< \mathbf{10^{-2}}$
	experiment 3	0.61	0.39	0.93	**0.025**
Germination to sporulation	strain 4000	1.27	0.75	2.15	0.36
	strain 876-1	1.06	0.63	1.77	0.83
	strain 747-4	0.56	0.33	0.95	**0.03**
	strain 40A	0.67	0.39	1.13	0.13
	strain 7.4.3301	0.81	0.48	1.35	0.42
	strain i1	0.60	0.35	1.04	0.07
	experiment 2	0.92	0.58	1.47	0.72
	experiment 3	0.73	0.48	1.12	0.15
Time to maturation	strain 4000	4.12	1.98	8.58	$< \mathbf{10^{-2}}$
	strain 876-1	3.34	1.60	6.98	$< \mathbf{10^{-2}}$
	strain 747-4	1.73	0.77	3.85	0.18
	strain 40A	1.92	0.88	4.16	0.10
	strain 7.4.3301	1.29	0.57	2.95	0.54
	strain i1	0.65	0.25	1.71	0.38
	experiment 2	0.24	0.12	0.49	$< \mathbf{10^{-2}}$
	experiment 3	0.35	0.19	0.63	$< \mathbf{10^{-2}}$
From first chasmothecia to maturation	strain 4000	2.41	1.15	5.06	**0.025**
	strain 876-1	2.23	1.07	4.67	**0.03**
	strain 747-4	1.44	0.64	3.27	0.38

Table 10: The estimated relative rates and their 95% confidence intervals for the different studied infection event times, together with the associated test statistic for rejecting the null hypothesis of the corresponding factor having no effect on the rate of the event. Significant deviations are shown in bold.

Event	Factor	exp.coef.	lower95.	upper95.	p-value
	strain 40A	2.64	1.21	5.76	**0.025**
	strain 7.4.3301	1.47	0.64	3.39	0.36
	strain i1	0.91	0.34	2.40	0.85
	experiment 2	0.38	0.20	0.73	$< \mathbf{10^{-2}}$
	experiment 3	0.83	0.47	1.45	0.50

7 Abundance models

7.1 Pairwise model comparisons for abundance models

Table 11: The results from pairwise model comparisons, for the ordinal regression models where the model with only experiment id and the same model with both the experiment id and strain id as predictors are contrasted using anova. The presented p-value corresponds to the evidence in favor of the more rich model (Model 2).

Process	log-likelihood Model 1	log-likelihood Model 2	P(>|Chi|)
Bevan scale at day 15	-148	-140	$\mathbf{0.01}$
The immature chasmothecia category	-252	-232	$< \mathbf{10^{-2}}$

Table 12: The results from pairwise model comparisons, for the ordinal regression models where the model with only strain id and the same model with both the experiment id and strain id as predictors are contrasted using anova. The presented p-value corresponds to the evidence in favor of the more rich model (Model 2).

Process	log-likelihood Model 1	log-likelihood Model 2	P(>|Chi|)
Bevan scale at day 15	-155	-140	$< \mathbf{10^{-2}}$
The immature chasmothecia category	-236	-232	$\mathbf{0.0213}$

7.2 Bevan scale at day 15

Table 13: Estimated effects for the ordinal regression model for the Bevan scale at day 15. Significant deviations are shown in bold.

	Estimate	Std. Error	z value	p-value
strain 4000	0.83	0.66	1.24	0.21
strain 876-1	0.87	0.64	1.36	0.17
strain 747-4	1.02	0.60	1.71	0.09
strain 40A	-0.24	0.59	-0.41	0.68
strain 7.4.3301	0.68	0.64	1.07	0.29
strain i1	-1.33	0.69	-1.93	**0.05**
First sporulation	-0.77	0.09	-8.34	$\mathbf{< 10^{-2}}$
experiment 2	0.98	0.80	1.23	0.22
experiment 3	-1.60	0.64	-2.50	**0.01**

7.3 Final immature chasmothecia category

Table 14: Estimated effects for the ordinal regression model for the (immature) chasmothecia category by the end of the follow-up.

	Estimate	Std. Error	z value	p-value
strain 4000	0.9896	0.5989	1.6524	0.098
strain 876-1	1.9504	0.6066	3.2155	$\mathbf{< 10^{-2}}$
strain 747-4	1.2732	0.6020	2.1149	**0.034**
strain 40A	0.8032	0.6107	1.3152	0.188
strain 7.4.3301	1.1254	0.6059	1.8574	0.063
strain i1	0.1238	0.6593	0.1877	0.851
First chasmothecia	-0.4612	0.0455	-10.1461	$\mathbf{< 10^{-2}}$
experiment 2	-0.4325	0.4777	-0.9054	0.365
experiment 3	-0.0300	0.4314	-0.0696	0.944

8 Life-history trait correlations

To systematically assess how different life-history times are correlated, and the possibility of trade-offs among them, we considered all pairwise models, where another measured trait, together with strain- and experiment identity as additional predictors, were considered as predictors. Tables 15, 16 and 17 collect all the estimated effects for the predictors in the columns and the estimated statistical significancy of them when predicting the outcome indicated in the table rows. Whenever the predicted outcome is an event-time, then a cox proportional hazards survival model is used. For the survival models the relative rate (i.e. the exponential of the estimated coefficient is reported, and thus a positive value indicates positive correlation in event times, which corresponds to positive correlations in the rates of acquiring these stages.

For the abundance measures the ordinal regression model is utilized, and there we report the estimated coefficient. As there the response variable is infection abundance, while the predictor is an event time, there a negative coefficient means that later accomplished life-history stages coincides with lesser abundance. This correponds to positive correlation between speed and abundace.

predicted trait	Germination	Sporulation	Chasmothecia	Mature chasmothecia	From germination to sporulation	From sporulation to chasmothecia	From chasmothecia to mature
Sporulation	0.70($<\mathbf{10^{-2}}$)	NA	NA	NA	NA	NA	NA
Chasmothecia	0.59($<\mathbf{10^{-2}}$)	0.74($<\mathbf{10^{-2}}$)	NA	NA	NA	NA	NA
Mature chasmothecia	0.53($<\mathbf{10^{-2}}$)	0.77($<\mathbf{10^{-2}}$)	0.81($<\mathbf{10^{-2}}$)	NA	NA	NA	NA
From germination to sporulation	0.97(0.559)	NA	NA	NA	NA	NA	NA
From sporulation to chasmothecia	0.70($<\mathbf{10^{-2}}$)	0.88($<\mathbf{10^{-2}}$)	NA	NA	0.98(0.47)	NA	NA
From chasmothecia to mature	0.84(0.14)	0.99(0.87)	1.00(0.57)	NA	1.04 (0.47)	1.03 (0.29)	NA

Table 15: The estimated relative rates (exponentials of the estimated coefficients) for using the columns as predictor when predicting the event times indicated by the rows. The statistical significancy of the estimated effect is shown in parenthesis and the significant effects are shown in bold. With NA's we have omitted the pairs of events occurring in wrong order, as they lead to non-intuitive modelling, as well as

predicted trait	Germination	Sporulation	Chasmothecia	Mature chasmothecia	From germination to sporulation	From sporulation to chasmothecia	From chasmothecia to mature
Bevan scale at day 15	-0.83 ($<\mathbf{10^{-2}}$)	-0.77 ($<\mathbf{10^{-2}}$)	-0.47 ($<\mathbf{10^{-2}}$)	-0.37 ($<\mathbf{10^{-2}}$)	-0.05 (0.338)	-0.24 ($<\mathbf{10^{-2}}$)	0.70 (0.47)
Final chasmothecia category	-0.67 ($<\mathbf{10^{-2}}$)	-0.43 ($<\mathbf{10^{-2}}$)	-0.41 ($<\mathbf{10^{-2}}$)	-0.36 ($<\mathbf{10^{-2}}$)	-0.30 ($<\mathbf{10^{-2}}$)	-0.32 ($<\mathbf{10^{-2}}$)	-0.10 (**0.05**)

Table 16: The estimated effects of event timings (columns) as predictor when predicting the abundance measureds indicated by the rows. The statistical significancy of the estimated effect is shown in parenthesis and the significant effects are shown in bold.

predicted trait	Germination	Sporulation	Chasmothecia	Mature chasmothecia	From germination to sporulation	From sporulation to chasmothecia	From chasmothecia to mature
Time to A1	0.84 ($<\mathbf{10^{-2}}$)	0.80 ($<\mathbf{10^{-2}}$)	0.90 ($<\mathbf{10^{-2}}$)	0.89 ($<\mathbf{10^{-2}}$)	0.89 ($<\mathbf{10^{-2}}$)	1.05 (0.04)	1.05 (0.05)
Time to A2	0.87 ($<\mathbf{10^{-2}}$)	0.86 ($<\mathbf{10^{-2}}$)	0.94 ($<\mathbf{10^{-2}}$)	0.91 ($<\mathbf{10^{-2}}$)	0.91 ($<\mathbf{10^{-2}}$)	1.04 (0.12)	1.03 (0.31)
From sporulation to A1	0.94 (0.13)	0.98 (0.54)	1.01 (0.70)	0.97 (0.26)	0.97 (0.26)	1.03 (0.31)	0.96 (0.15)
From sporulation to A2	1.01 (0.84)	1.10 (**0.02**)	1.05 (**0.04**)	0.99 (0.65)	0.99 (0.65)	1.03 (0.37)	0.94 (**0.01**)

Table 17: The estimated relative rates (exponentials of the estimated coefficients) for using event times in the columns as predictor for the hyperparasite infection event times, indicated by the rows. The statistical significancy of the estimated effect is shown in parenthesis and the significant effects are shown in bold.

9 Survival model fits for the hyperparasite

9.1 Pairwise model comparisons for the timings of hyperparasite infection stages

Table 18: The results from pairwise model comparisons, for the survival models presented in where a survival model without any predictors and a model with the strain as a predictor are contrasted using anova. The presented p-value corresponds to the evidence in favor of the more rich model (Model 2).

Process	log-likelihood Model 1	log-likelihood Model 2	P(>|Chi|)
Time to A1	-470.94	-464.77	$\mathbf{0.05}$
Time from mildew sporulation to A1	-455.65	-446.26	$< \mathbf{10^{-2}}$
Time to A2	-321.96	-313.32	$< \mathbf{10^{-2}}$
Time from mildew sporulation to A2	-314.45	-301.07	$< \mathbf{10^{-2}}$

Table 19: The results from pairwise model comparisons, for survival models where a survival model with strain id as a predictor and a model with the strain and pathogen infection status at day 8 as a predictor are contrasted using anova. The presented p-value corresponds to the evidence in favor of the more rich model (Model 2).

Process	log-likelihood Model 1	log-likelihood Model 2	P(>|Chi|)
Time to A1	-464.77	-446.61	$< \mathbf{10^{-2}}$
Time from mildew sporulation to A1	-446.26	-445.26	0.37
Time to A2	-313.32	-306.12	$< \mathbf{10^{-2}}$
Time from mildew sporulation to A2	-301.07	-300.36	0.49

Table 20: The results from pairwise model comparisons, for the survival models where a survival model with pathogen infection status at day 8 as a predictor and a model with the strain id and pathogen infection status at day 8 as a predictor are contrasted using anova. The presented p-value corresponds to the evidence in favor of the more rich model (Model 2).

Process	log-likelihood Model 1	log-likelihood Model 2	P(>|Chi|)
Time to A1	-457.08	-446.61	$< \mathbf{10^{-2}}$
Time from mildew sporulation to A1	-454.93	-445.26	$< \mathbf{10^{-2}}$
Time to A2	-316.97	-306.12	$< \mathbf{10^{-2}}$
Time from mildew sporulation to A2	-313.55	-300.36	$< \mathbf{10^{-2}}$

9.2 Timings of hyperparasite infection stages

9.2.1 Without the pathogen infection status as a predictor

Table 21: The estimated relative rates and their 95% confidence intervals for the different studied hyperparasite infection event times, together with the associated test statistic for rejecting the null hypothesis of the corresponding factor having no effect on the rate of the event. Significant deviations are shown in bold.

Event	predictor	coef	exp.coef.	lower95.	upper95.	p-value
Time to A1	strain 4000	0.45	0.77	0.39	1.52	0.4508
	strain 876-1	0.07	0.52	0.26	1.06	0.0730
	strain 747-4	0.01	0.37	0.18	0.77	$\mathbf{< 10^{-2}}$
	strain 40A	0.01	0.40	0.20	0.81	**0.0107**
	strain 7.4.3301	0.08	0.54	0.28	1.07	0.0760
	strain i1	0.01	0.37	0.18	0.76	$\mathbf{< 10^{-2}}$
Time from mildew sporulation to A1	strain 4000	0.048	1.05	0.95	2.08	0.90
	strain 876-1	-0.86	0.42	0.21	0.86	**0.017**
	strain 747-4	-1.04	0.35	0.17	0.73	**0.005**
	strain 40A	-1.007	0.37	0.18	0.75	**0.006**
	strain 7.4.3301	-0.26	0.77	0.39	1.52	0.45
	strain i1	-0.56	0.57	0.27	1.17	0.13
Time to A2	strain 4000	0.39	1.39	0.65	2.98	0.390
	strain 876-1	0.05	0.39	0.15	1.00	**0.050**
	strain 747-4	0.04	0.38	0.15	0.95	**0.040**
	strain 40A	0.03	0.33	0.12	0.87	**0.025**
	strain 7.4.3301	0.49	0.76	0.35	1.66	0.491
	strain i1	0.21	0.58	0.25	1.35	0.208
Time from mildew sporulation to A2	strain 4000	0.73	2.08	0.97	4.47	**0.05**
	strain 876-1	-0.97	0.38	0.14	0.96	**0.041**
	strain 747-4	-0.97	0.38	0.15	0.96	**0.04**
	strain 40A	-1.16	0.31	0.12	0.84	**0.02**
	strain 7.4.3301	-0.06	0.95	0.42	2.06	0.88
	strain i1	-0.11	0.88	0.38	2.06	0.78

9.2.2 With the pathogen infection status as a predictor

Table 22: The estimated relative rates and their 95% confidence intervals for the different studied hyperparasite infection event times, where in the model the pathogen infection stage at day 8 was accounted for, together with the associated test statistic for rejecting the null hypothesis of the corresponding factor having no effect on the rate of the event. Significant deviations are shown in bold.

Event	predictor	coef	exp.coef.	lower95.	upper95.	p-value
Time to A1	strain 4000	-0.0301	0.9704	0.47	1.96	0.933486
	strain 876-1	-1.0450	0.35	0.17	0.72	$\mathbf{< 10^{-2}}$
	strain 747-4	-1.0542	0.34	0.16	0.71	$\mathbf{< 10^{-2}}$
	strain 40A	-1.2569	0.28	0.13	0.58	$\mathbf{< 10^{-2}}$
	strain 7.4.3301	-0.6760	0.50	0.25	1.00	$\mathbf{0.05}$
	strain i1	-1.0332	0.35	0.17	0.73	$\mathbf{< 10^{-2}}$
	Bevan 1	1.1686	3.21	1.69	6.09	$\mathbf{< 10^{-2}}$
	Bevan 2	2.8315	16.97	6.96	41.3628	$\mathbf{< 10^{-2}}$
Time from mildew sporulation to A1	strain 4000	0.07	1.07	0.53	2.15	0.83
	strain 876-1	-0.94	0.38	0.18	0.8	$\mathbf{0.01}$
	strain 747-4	-1.02	0.36	0.17	0.74	$\mathbf{< 10^{-2}}$
	strain 40A	-1.04	0.35	0.17	0.72	$\mathbf{< 10^{-2}}$
	strain 7.4.3301	-0.3	0.73	0.37	1.46	0.38
	strain i1	-0.59	0.55	0.26	1.15	0.11
	Bevan 1	0.23	1.26	0.66	2.41	0.47
	Bevan 2	0.60	1.83	0.78	4.26	0.15
Time to A2	strain 4000	0.40	1.50	0.67	3.34	0.31
	strain 876-1	-1.16	0.31	0.12	0.80	$\mathbf{0.015}$
	strain 747-4	-0.96	0.38	0.14	0.97	0.04
	strain 40A	-1.26	0.28	0.10	0.75	$\mathbf{0.012}$
	strain 7.4.3301	-0.27	0.75	0.34	1.66	0.49
	strain i1	-0.54	0.58	0.25	1.34	0.2
	Bevan 1	0.92	2.51	1.15	5.46	$\mathbf{0.02}$
	Bevan 2	1.81	6.14	2.38	15.83	$\mathbf{< 10^{-2}}$
Time from mildew sporulation to A2	strain 4000	0.72	2.05	0.94	4.47	0.07
	strain 876-1	-1.03	0.35	0.13	0.91	$\mathbf{0.03}$
	strain 747-4	-0.94	0.38	0.15	0.99	$\mathbf{0.05}$
	strain 40A	-1.17	0.3	0.11	0.82	$\mathbf{0.02}$
	strain 7.4.3301	-0.09	0.91	0.41	2.00	0.81
	strain i1	-0.13	0.87	0.37	2.04	0.75
	Bevan 1	0.2	1.22	0.55	2.68	0.62
	Bevan 2	0.56	1.75	0.67	4.53	0.24

10 Fitness traits and metapopulation prevalence

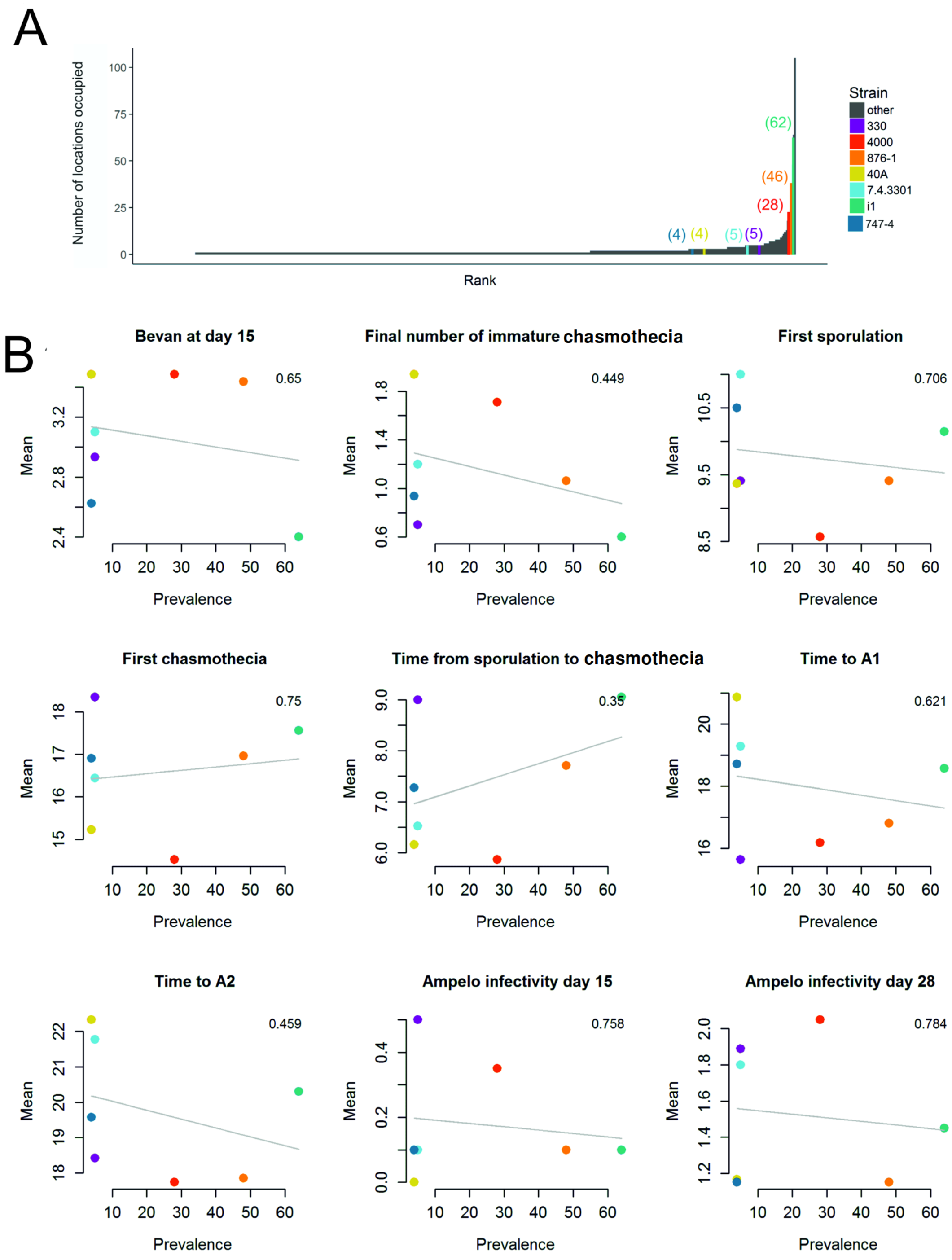

Figure 5: Panel A shows the frequency distribution for the number of occupied locations for all the observed strains in 2015. The majority of observed strains (395) in that year were only found in a single location, but 3 strains were found in >25 discrete locations. Strains used to study life-history variation are shown in color. In panel B the prevalence of strains across the metapopulation is correlated with the mean fitness traits, and the corresponding p-values for the fitted rank correlations are shown in upright.

11 Bibliography

References

[1] Bevan J.R., Crute I.R., Clarke D.D. (1993). Diversity and variation in expression of resistance to Erysiphe-fischeri in Senecio-vulgaris. Plant Pathology, 42, 647–653.

[2] Falk S.P., Gadoury D.M., Pearson R.C., Seem R.C. (1995). Partial central of grape powdery mildew by the mycoparasite Ampelomyces- quisqualis. Plant Disease, 79, 483–490.

[3] Tollenaere, C., Pernechele, B., Mäkinen, H., Parratt, S., Németh, M., Kovács, G. et al. (2014). A hyperparasite affects the population dynamics of a wild plant pathogen. Molecular Ecology, 23, 5877-5887.
